# A Rare Presentation of Small Bowel Angiomyolipoma: A Case Report

**DOI:** 10.7759/cureus.92041

**Published:** 2025-09-11

**Authors:** Aneesh Kumar, Ujjwal Sikhar, Mumtaz A Ansari, Arvind Pratap, Vivek Srivastava

**Affiliations:** 1 Department of General Surgery, Institute of Medical Sciences, Banaras Hindu University, Varanasi, IND

**Keywords:** angiomyolipoma, bowel obstruction, complicated inguinal hernia, herniorrhaphy, small bowel tumor

## Abstract

Angiomyolipoma (AML) of the small bowel is a rare entity. It usually presents with intestinal obstruction, gastrointestinal hemorrhage, or intussusception. However, sometimes it may have an unusual presentation, posing intraoperative challenges. Here we report an unusual presentation of this rare disease.

A 32-year-old male presented to the emergency department following an abandoned irreducible right inguinal hernia repair at a peripheral hospital. The patient had a transected segment of small bowel extruding from the incision site, sutured to the skin margin. On assessment, a 6x2 cm mass was discovered arising from the transected segment of the extruding bowel. After a formal exploratory laparotomy, a segment of small bowel, including the mass and transected bowel, was resected and brought out as a double-barrel ileostomy in the right iliac fossa. The right inguinal hernia was repaired with Bassini’s herniorrhaphy. Histopathological examination confirmed the mass lesion as an AML.

This case underlines the necessity of a comprehensive preoperative assessment and knowledge of uncommon intra-abdominal pathology. Proper diagnosis and surgical planning are required to prevent intraoperative complications and enable total excision, thereby minimizing the chance of recurrence.

## Introduction

Angiomyolipomas (AMLs) are rare benign mesenchymal hamartomas comprising varying proportions of blood vessels, smooth muscle cells, and adipose tissue. They are mostly located in the kidney and are often associated with tuberous sclerosis complex (TSC), in which they may present as multiple and bilateral lesions [1]. Extrarenal AMLs are extremely uncommon, with the liver being the most frequently reported extrarenal site in the literature [1].

Cases of small intestine AMLs are rare, and involvement of the gastrointestinal (GI) system is very uncommon. These tumors may lead to numerous clinical manifestations depending on their location and size, including intestinal obstruction, anemia, diarrhea, abdominal pain, melena, or intussusception [2]. The majority of cases are discovered intraoperatively or through histological analysis, and preoperative diagnosis is sometimes difficult because of vague clinical and radiological characteristics. Since inadequate resection increases the chance of recurrence, total surgical excision remains the preferred treatment [3]. Because these cases are uncommon and diagnostically challenging, it is crucial to describe them to raise awareness and add to the body of existing literature. Here, we present a rare case of small bowel AML complicating an inguinal hernia repair, an uncommon clinical scenario that is rarely reported. This case report has been prepared in accordance with the SCARE checklist [4].

## Case presentation

A 32-year-old male presented to the emergency room after referral from a private hospital following an aborted surgical procedure (open hernia repair) for a right indirect irreducible hernia performed six hours earlier. The operative site was dressed, and the operative note mentioned an inadvertent enterotomy of the small bowel. There was no significant family history.

**Figure 1:**
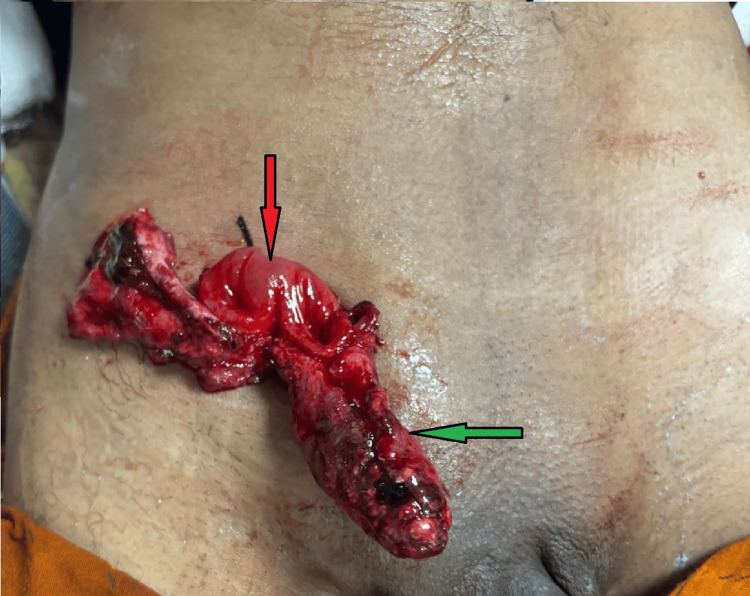
Transected bowel loop sutured to the skin (red arrow) and the attached mass lesion (green arrow).

On clinical examination, he had a pulse rate of 106 beats per minute, blood pressure of 120/72 mmHg, respiratory rate of 20 breaths per minute, and oxygen saturation of 100% on room air. On abdominal examination, there was tenderness in the right iliac fossa, and the operative site revealed a completely transected segment of small bowel. The transected ends were sutured to the wound margin, and a 6×2 cm mass was seen attached to one of the extruded bowel segments (Figure 1).

**Figure 2:**
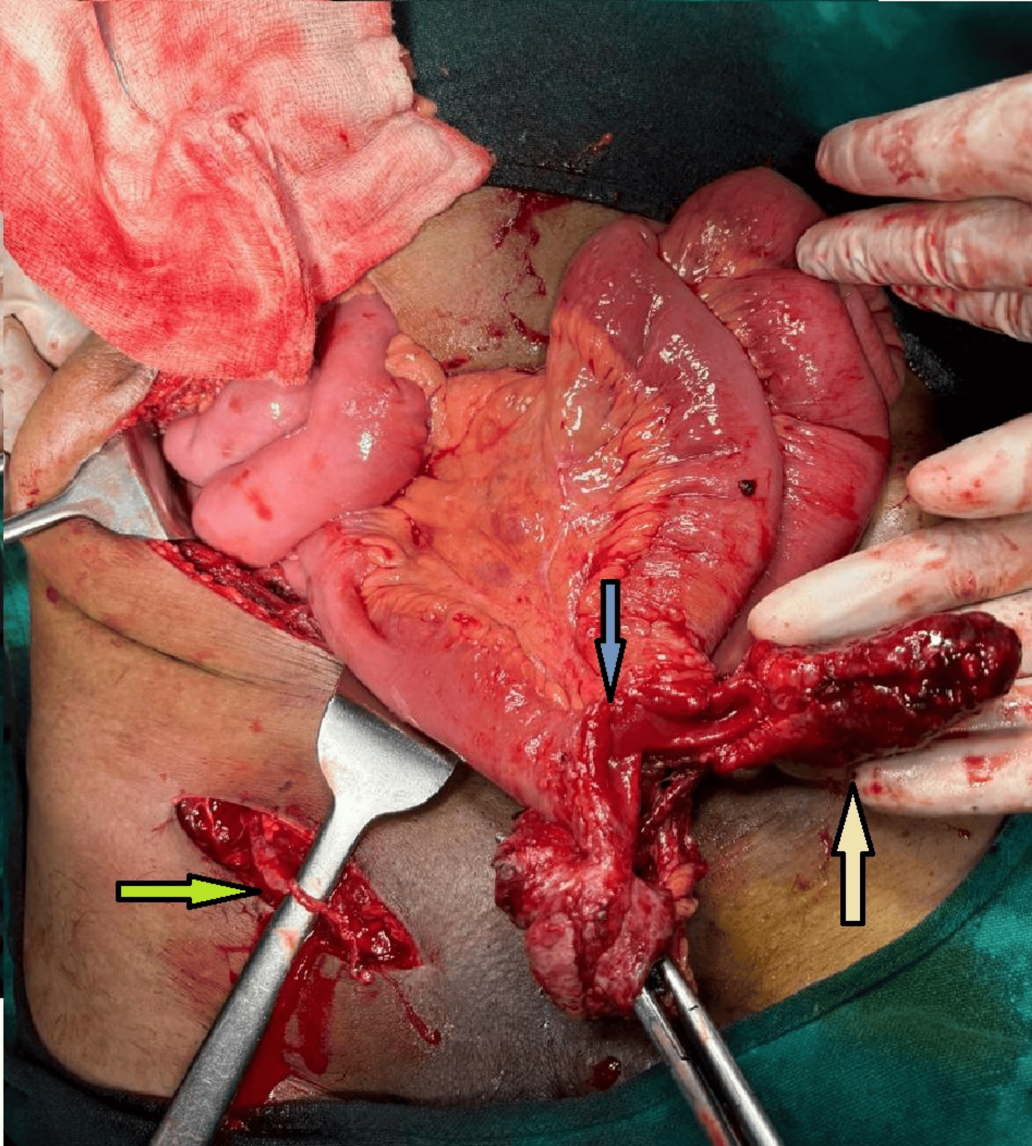
Completely transected ileal loop (blue arrow) with the attached mass lesion on the proximal end (white arrow). The yellow arrow indicates the incision site in the right inguinal region.

The patient was resuscitated and hemodynamically stabilized in the emergency room upon arrival. He was then shifted to the operating theater, where an emergency exploratory laparotomy was performed. Intraoperatively, after removal of the skin sutures, the bowel was reduced back through the previous operative site in the right inguinal region. A complete transection of the ileal loop was found approximately 2 feet proximal to the ileocecal junction. A firm-to-hard mass was attached to the proximal segment of the transected bowel (Figure 2).

**Figure 3:**
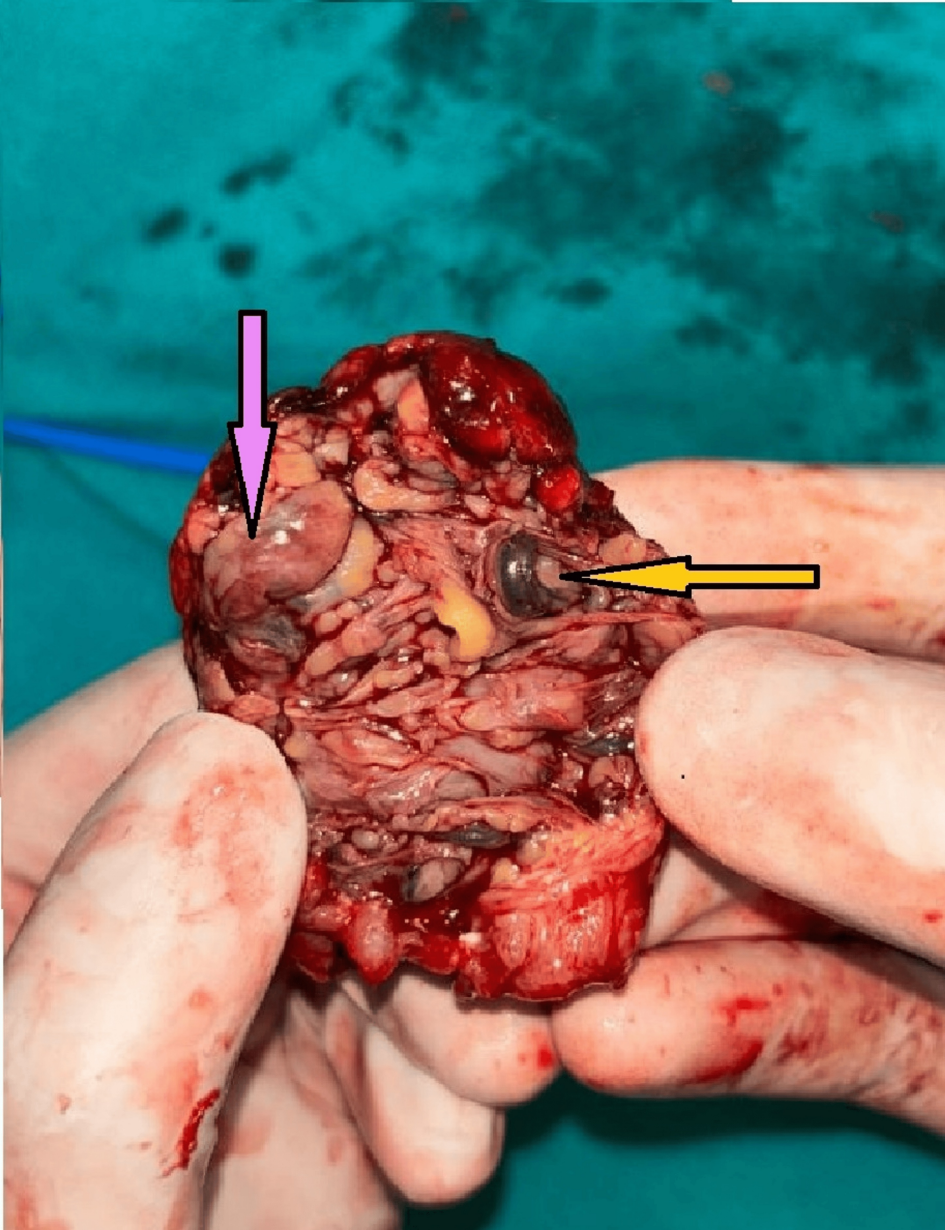
Cut section of the mass lesion, revealing adipose tissue (purple arrow) and blood vessels (yellow arrow) within it.

**Figure 4:**
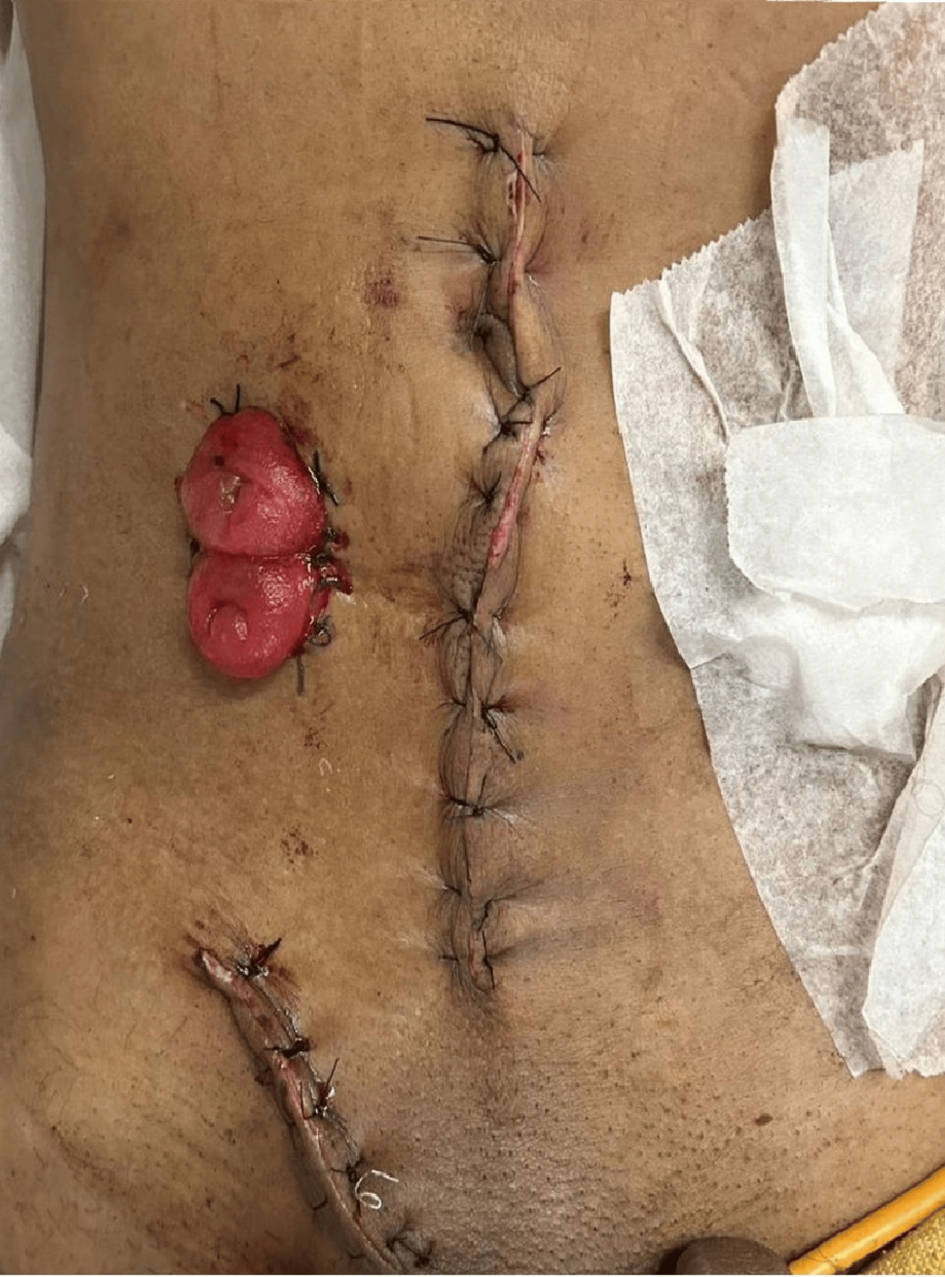
Incision lines for the midline exploratory laparotomy and right inguinal hernia repair, along with the double-barrel ileostomy in the right iliac fossa.

The involved bowel segment, along with the mass lesion, was resected (Figure 3). As the bowel appeared inflamed, both ends of the ileal loop were exteriorized as a double-barrel ileostomy in the right iliac fossa. In addition, a modified Bassini’s herniorrhaphy was performed for the right inguinal hernia (Figure 4).

**Figure 5:**
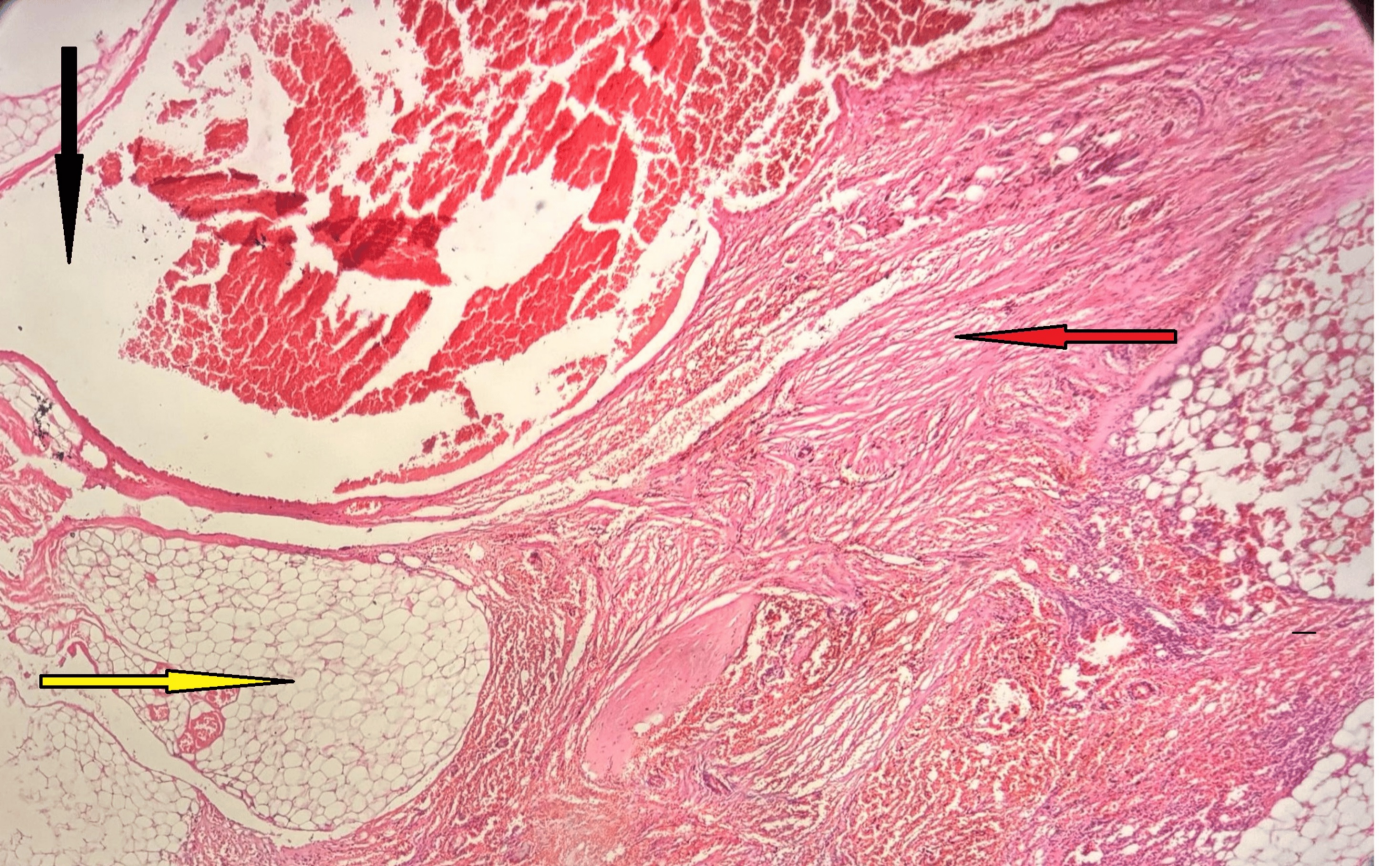
Histopathological image showing features of AML. The section demonstrates dilated and congested blood vessels (black arrow), mature adipocytes (yellow arrow), and bundles of smooth muscle cells (red arrow). AML, angiomyolipoma

The stoma became functional on postoperative day 2, and oral intake was initiated the same day, which he tolerated well. He was discharged on the fourth postoperative day with appropriate advice. Histopathological examination of the resected specimen revealed features consistent with AML (Figure 5). Immunohistochemical staining demonstrated positivity for alpha smooth muscle actin (SMA), desmin, and vimentin.

## Discussion

AML is a benign mesenchymal tumor within the perivascular epithelioid cell (PEComa) family, composed of varying amounts of mature adipose tissue, smooth muscle, and thick-walled dysmorphic vasculature. Although the kidney is the primary site of AML, small-bowel involvement and extrarenal locations, such as the GI tract, are extremely rare. Preoperative diagnosis is frequently difficult because of nonspecific symptoms and overlap with more common benign lesions, such as lipomas or gastrointestinal stromal tumors (GIST), both radiologically and endoscopically.

Small bowel AMLs can present in various ways, with the location and size of the tumor playing a major role. Patients may present with anemia, intestinal obstruction, melena, intussusception, abdominal pain, or may remain asymptomatic until the tumor is found incidentally [5]. In this case, the tumor was discovered incidentally during inguinal hernia surgery involving a transected ileal loop. This unusual presentation highlights the diagnostic challenge.

The diagnosis can be suggested by imaging, showing macroscopic fat. Fat is detected by negative Hounsfield units on CT; when fat is microscopic, MRI usually demonstrates T1/T2 hyperintensity with signal suppression on fat-saturated sequences and characteristic chemical-shift effects [6]. Because imaging findings may overlap with other benign and malignant mesenchymal tumors, including lipomas, GISTs, and liposarcomas, radiological investigations often fail to provide a conclusive preoperative diagnosis for AML [7].

A GI lipoma, which is far more common (reported incidence across the gut ~0.035-4.4%, with the colon most frequently involved), is the main preoperative differential diagnosis for a fatty submucosal bowel lesion [8]. The presence of muscle and blood vessels in the tumor, in addition to fat cells, which constitute about 10-40%, helps distinguish it from intestinal lipomas, which have uniform fat content. Histopathological analysis remains the most reliable diagnostic method. Immunohistochemically, these tumors show positivity for HMB 45, vimentin, SMA, and desmin, while being negative for cytokeratin and CD-34 [9].

Complete surgical excision with clear margins is the recommended treatment for symptomatic or incidentally detected lesions with concerning features, both to resolve symptoms and to obtain a definitive diagnosis. Incomplete resection of the tumor increases the risk of recurrence [9]. This case contributes to the limited data regarding extrarenal gastrointestinal tract AMLs. In the differential diagnosis of small intestinal masses, it emphasizes the importance of considering uncommon benign tumors, particularly when they are discovered incidentally during unrelated surgical procedures.

## Conclusions

Small intestinal AMLs are uncommon tumors that often resemble other gastrointestinal lesions and are usually diagnosed only after histological confirmation. Comprehensive preoperative evaluation, including multidisciplinary consultation when necessary, detailed history taking and physical examination, and adequate radiographic assessment, is essential. This is a mandatory step in the diagnosis of rare diseases like AML, which may not be easily identified, particularly when they coincide with common surgical pathologies such as an inguinal hernia. Our case adds to the limited body of research and highlights the need for surgeons to be aware of uncommon presentations, such as small intestinal AML occurring in conjunction with other operative procedures. Early diagnosis, proper preoperative planning, and appropriate preoperative measures can all help ensure the best possible outcome and reduce the risk of recurrence or unexpected complications.
